# Evaluation of Nutritional Substances and Investigation of Antioxidant and Antimicrobial Potentials of *Boerhavia diffusa* with in Silico Molecular Docking

**DOI:** 10.3390/molecules27041280

**Published:** 2022-02-14

**Authors:** Mohandass Kaviya, Balamuralikrishnan Balasubramanian, Kathirvel Bharathi, Arunkumar Malaisamy, Naif Abdullah Al-Dhabi, Valan Arasu Mariadhas, Arumugam Vijaya Anand, Wenchao Liu

**Affiliations:** 1 Medical Genetics and Epigenetics Laboratory, Department of Human Genetics and Molecular Biology, Bharthiar University, Coimbatore 641046, India; kaviyamohandass@gmail.com (M.K.); bharathikathir30@gmail.com (K.B.); 2 Department of Food Science and Biotechnology, College of Life Science, Sejong University, Seoul 05006, Korea; bala.m.k@sejong.ac.kr; 3 Transcription Regulation Group, International Centre for Genetic Engineering and Biotechnology (ICGEB), New Delhi 110067, India; makias191@gmail.com; 4 Department of Botany and Microbiology, College of Science, King Saud University, P.O. Box 2455, Riyadh 11451, Saudi Arabia; naldhabi@ksu.edu.sa (N.A.A.-D.); mvalanarasu@gmail.com (V.A.M.); 5 College of Coastal Agricultural Sciences, Guangdong Ocean University, Zhanjiang 524088, China

**Keywords:** phytochemical, in vitro antioxidants, *Pseudomonas aeruginosa*, *Staphylococcus aureus*, molecular docking

## Abstract

*Boerhavia diffusa* L. Nyctanginaceae (*B. diffusa*) is a medicinal herb commonly considered as a weed. The exploration of phytochemicals in different parts of *B. diffusa* with different solvents will create awareness, along with the suitable solvent and method for extraction of pharmaceutical compounds. Hence, the present study focuses on phytochemical analysis of *B. diffusa* leaves, stems, and roots in various solvents with hot and cold extraction. The decoctions performed well in most of the qualitative and quantitative tests, along with the DPPH assay. The aqueous extract showed a good result in the FRAP assay and ABTS assay. In the antimicrobial test, the *B. diffusa* root ethanol extract inhibited the growth of *Pseudomonas aeruginosa* and *Staphylococcus aureus* with zones of inhibition of about 8 mm and 20 mm at 200 µg concentration, respectively. Using a molecular docking approach, the top four ranked molecules from the crude extract of *B. diffusa* profiled from GC–MS spectroscopy in terms of growth inhibition of the pathogenic bacterium *P. aeruginosa* were selected; among them, 2-(1,2 dihydroxyethyl)-5-[[2,5,7,8-tetramethyl-2-(4,8,12-trimethyltridecyl)-3,4-dihydrochromen-6-yl]oxy]oxolane-3,4-diol exhibited the minimum binding score, revealing high affinity in complex. *B. diffusa* is highly nutritious, and the maceration and decoction extracts were similar except for the chloroform extract that was found to be weak.

## 1. Introduction

Nature has been enriched with numerous medicinal plants whose medicinal properties are not much known or where the correct way of using the resource provided by nature is not known. The antioxidant properties of plants help in reducing oxidative stress [1]. *Staphylococcus aureus* and *Pseudomonas aeruginosa* are the bacterial types most commonly found to exhibit multidrug resistance [2]. Therefore, knowing the phytochemical profile of the plant, along with its antimicrobial and antioxidant properties in various parts, as a function of the extract and extraction procedure, can aid in the development of herbal medicine. The conventional process of drug development is complex, challenging, lavish, time-consuming, and exhausting. To overcome these barriers, computational approaches such as molecular docking have played a significant role in streamlining the road to drug development. The use of molecular docking technology in drug research is currently a valuable method for quickly screening candidates from drug libraries [3]. *B. diffusa* is commonly known as red spiderling or common hogweed. It belongs to the order Caryophyllales, indicating it to be a dicotyledonous herb or shrub. It belongs to the family Nyctanginaceae, indicating it to be a four o’clock plant. *B*. *diffusa* is an herb that branches laterally at ground level with green leaves, pinkish or purplish stems, and purple flowers that are campanulate [4,5]. About 40 species of *Boerhavia* are widely distributed throughout the tropical and subtropical regions of the world, with many variations in South and North America [6]. The plant grows in a creeping manner, and it has been found to grow more during the rainy season because the species *Boerhavia* tends to grow in wet soil and in sandy, stony, and clayey soils found near dried up water resources, as well as riverbeds, hill slopes, and mountains [7]. It is indigenous to India, where it is also referred to as Punarnava and used in traditional medicine [8]. In Tamil, the plant is identified by various terms such as Mukkuratai Kodi, Sarai, and Sarandai. The plant is enriched with anti-inflammatory [9], antibacterial [10] antioxidant [11], and immunomodulatory [12] properties. *B. diffusa* has been identified among many other plants to confer protection against SARS CoV-2 [13]. The secondary metabolites present in the plants can act as effective pharmaceutical compounds [14]. The antioxidant properties of the plants help in reducing oxidative stress in humans [15]. Quercetin which is a flavonoid compound obtained from the plant can be used as a treatment for coronary heart disease and fatty liver [16]. The volatile compounds have been identified to have a variety of pharmaceutical roles when consumed in the form of aroma and fresh juice with anti-inflammatory, antimicrobial, antioxidant, anti-depressive, and analgesic properties [17,18,19]. Quorum sensing is an important behavior exhibited by bacteria that regulates gene expression on the basis of the cell density and signals produced, which control the behavior of the group [20]. Quorum sensing has also been found to be responsible for the virulence, amplification, and antimicrobial resistance of bacteria; hence, quorum-sensing inhibitors are of recent interest for antimicrobial activity. In a study conducted with *P. aeruginosa,* PqsR was found to be a useful receptor for quorum-sensing inhibition [21]. The present study conducts an in vitro antioxidant and antimicrobial analysis of different parts of *B. diffusa* (leaves, stems, and roots) and their phytochemicals in various extracts (decoction, maceration extracts using aqueous, ethanol, and chloroform). Additionally, the phytochemicals extracted from the crude extract of *B. diffusa* were exploited against pathogenic bacteria through a molecular docking experiment using GLIDE ligand docking, Schrodinger software. The phytochemical analysis involved qualitative tests for carbohydrates, proteins, amino acids, phenols, flavonoids, terpenoids, phytosterols, saponins, and quinone. It also involved the quantification of chlorophyll, carbohydrates, proteins, amino acids, phenols, and flavonoids. The in vitro antioxidant analysis constituted DPPH, FRAP, and ABTS assays. The antimicrobial activity against *P. aeruginosa* and *S. aureus* was determined using the disc diffusion method. The extract that showed good results in the antimicrobial tests was subjected to GC–MS analysis to qualitatively identify the presence of volatile compounds that could have antimicrobial potential. The results revealed that *B. diffusa* leaves are highly nutritious compared to other parts, whereas the roots provided the most substantial antibacterial activity against *P. aeruginosa* and *S. aureus.* The extraction procedure involving hot and cold extraction did not show much variance, except that the ethanolic extract performed well in the antimicrobial tests, whereas the chloroform extract did not. 

## 2. Results

### 2.1. Qualitative Tests

In the qualitative tests, the aqueous extract and decoctions of *B. diffusa* leaves, stems, and roots showed the presence of most phytochemicals such as proteins, flavonoids, terpenoids, carbohydrates, phenols, and phytosterols. The chloroform extracts of *B. diffusa* leaves, stems, and roots unanimously showed the presence of proteins, while phenol was present in *B. diffusa* leaves and stems. The ethanolic extract of *B. diffusa* leaves, stems, and roots showed the presence of carbohydrates, amino acids, and alkaloids, along with terpenoids in roots and stems, as well as phenol in stems and leaves. The *B. diffusa* root ethanolic extract also showed the presence of flavonoids. The decoctions of *B. diffusa* stood out among all other extracts, while *B. diffusa* aqueous extracts were similar to the decoctions. Ethanolic extracts showed the presence of alkaloids which were mostly absent in decoctions. The chloroform extract showed the presence of the least number of phytochemicals. The results are presented in Table 1, where + indicates the presence of the compound, ++ indicates a higher presence, and − indicates the absence of the compound.

### 2.2. Quantitative Tests

- Chlorophyll

The chlorophyll content was measured in the fresh leaves and stems of *B. diffusa*. The chlorophyll content was found to be higher in the leaves of *B. diffusa* than the stems. The chlorophyll content is expressed in mg/g as the mean ± standard error (SE) in Table 2.

- Carbohydrates

Carbohydrate levels were found to be high in the decoctions of *B. diffusa* leaves and stems. The carbohydrate content was high in the aqueous extract of the stem. Maximum carbohydrate content was observed in the *B. diffusa* root decoction. The carbohydrate content in *B. diffusa* leaves, stems, and roots is expressed in mg/g as the mean **±** SE in Table 3.

- Proteins

In the total protein estimation, the *B. diffusa* decoction showed the highest protein concentration of about 305.8 ± 5.8 mg/g. The aqueous extract of the *B. diffusa* leaves was the next highest, and the same pattern was observed in the *B. diffusa* stem decoction and aqueous extract. The *B. diffusa* roots showed the highest protein concentration among chloroform extracts. The results are expressed in mg/g as the mean ± SE in Table 4.

- Amino Acids

The *B. diffusa* leaf decoction and stem ethanolic extract showed the maximum amino-acid content. The ethanolic extracts showed close values to the decoction, whereas the root ethanolic extract showed the maximum value of amino acids. The results of amino-acid content in the *B. diffusa* leaves, stems, and roots are expressed in mg/g as the mean ± SE in Table 5.

- Flavonoids

The flavonoid content was found to be high in the decoctions of *B. diffusa* stems and roots, while the aqueous leaf extract showed a high level of flavonoids. The *B. diffusa* leaves were observed to have the highest flavonoid content among all extracts. The flavonoid content observed in the *B. diffusa* leaves, stems, and roots are expressed in mg/g as the mean ± SE in Table 6.

- Phenols

The phenolic content was found to be high in the decoctions of *B. diffusa* stems and roots, while the aqueous leaf extract showed a slightly greater level of phenolic content. The *B. diffusa* leaves were observed to have the highest phenolic content among all extracts. The phenolic contents are expressed in mg/g as the mean ± SE in Table 7.

### 2.3. In Vitro Antioxidant Assays

- DPPH Assay

**Figure 1:**
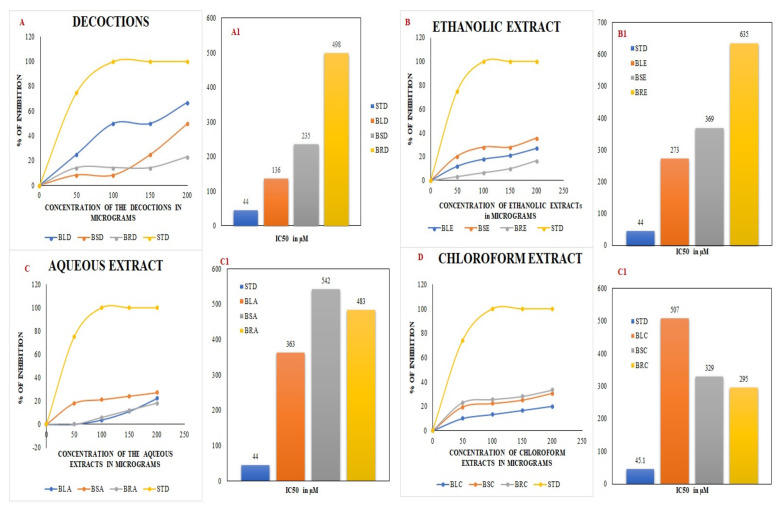
DPPH assay using decoctions (**A**) and ethanolic (**B**), aqueous (**C**), and chloroform (**D**) extracts of *B. diffusa.* (**A1**–**D1**) depict the IC_50_ values obtained from the respective DPPH assays. BLD, BSD, and BRD are the decoctions of *B. diffusa* leaves, stems, and roots. The ethanolic extracts of *B. diffusa* leaves, stems, and roots are denoted as BLE, BSE, and BRE. The aqueous and chloroform extracts of *B. diffusa* leaves, stems, and roots are denoted as BLA, BSA, BRA, BLC, BSC, and BRC respectively. The values expressed in the graph are the means of triplicate samples. STD in the figure denotes the standard ascorbic acid.

The decoctions of the *B. diffusa* leaves and stems, as well as the ethanol extracts, showed the maximum scavenging activity against DPPH. The *B. diffusa* leaves showed the highest inhibition among decoctions. The IC_50_ values were calculated from the inhibitory concentrations of each extract using the trend line equation. The inhibitory concentrations are shown as the percentage inhibition for concentrations from 0–200 µg, along with their IC_50_ values, in Figure 1.

#### 2.3.1. FRAP Assay

**Figure 2:**
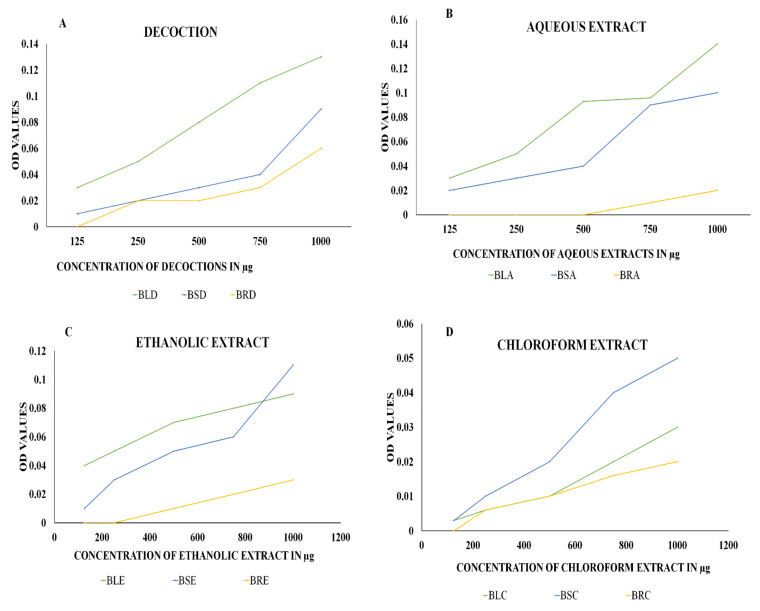
**The** FRAP assay using (**A**) decoctions and (**B**) aqueous, (**C**) ethanolic, and (**D**) chloroform extracts of *B. diffusa.* The figures represent the reducing power of the extracts against ferric chloride. An increase in OD values represents increasing reducing power of the extracts with an increase in concentration measured at 700 nm. BLD, BSD, and BRD are the decoctions of *B. diffusa* leaves, stems, and roots. The ethanolic extracts of *B. diffusa* leaves, stems, and roots are denoted as BLE, BSE, and BRE. The aqueous and chloroform extracts of *B. diffusa* leaves, stems, and roots are denoted as BLA, BSA, BRA, BLC, BSC, and BRC, respectively.

The decoctions of the leaves, stems, and roots showed the maximum reducing power in the FRAP assay. The *B. diffusa* leaves showed the highest power among aqueous extracts, with lower reduction levels found in the chloroform extracts. The OD values obtained for the extracts in the concentration range 125–1000 µg are depicted in Figure 2.

#### 2.3.2. ABTS Assay

**Figure 3:**
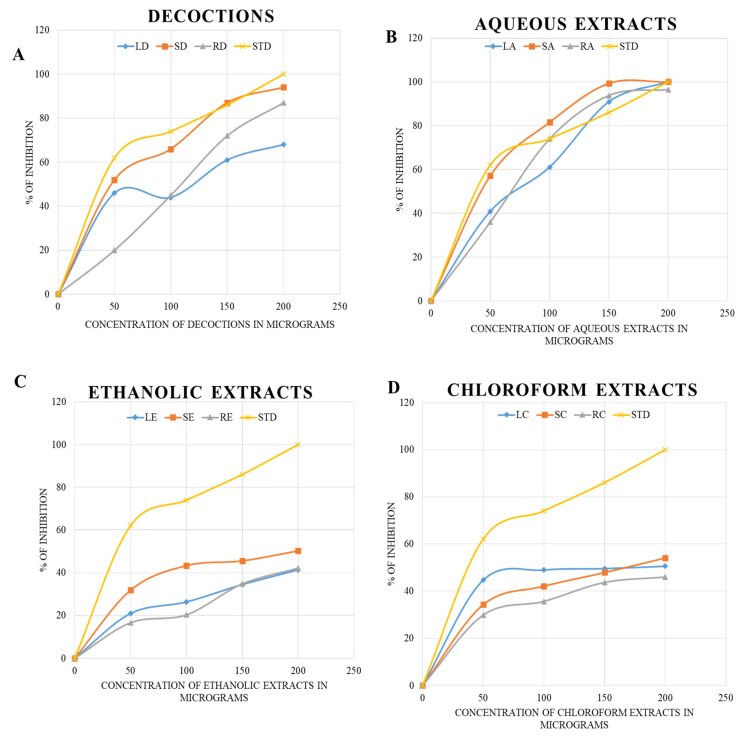
The ABTS assay using decoctions (**A**) and aqueous (**B**), ethanolic (**C**), and chloroform (**D**) extracts of *B. diffusa.* The LD, SD and RD represents the decoction of leaf, stem and root respectively. The LA, SA, RA, LE, SE and RE represents the aqueous and ethanolic extracts. The chloroform extracts of leaf, stem and root are given as LC. SC and RC respectively. The STD refers to the standard used for ABTS assay.

In the ABTS assay, the maximum level of inhibition (about 100%) was achieved in the aqueous extracts of *B. diffusa* leaves and stems at 200 and 150 µg concentrations, respectively. The aqueous extracts and decoctions were similar to the standard ascorbic acid. The inhibitory concentrations are shown as the percentage inhibition for concentrations 0–200 µg in Figure 3.

### 2.4. Antimicrobial Activity

Antimicrobial activity was detected in the *B. diffusa* decoctions of leaves, stems, and roots, as well as the ethanolic extracts of leaves and roots. The aqueous and chloroform extracts did not show any antimicrobial resistance. The maximum inhibition of *P. aeruginosa* (ATCC 27853) was found in the decoctions of leaves and stems, as well as the ethanolic extract of roots. The maximum inhibition of bacterial growth was found at 200 µg concentration of the extracts, with a zone of about 8 mm. The zone of inhibition of various extracts is presented in mm as the mean ± standard error in Table 8.

In terms of the antimicrobial activity against *S. aureus* (ATCC 25923), all extracts showed antibacterial activity except for the chloroform stem extract. The chloroform extract of leaves and roots showed inhibition zones of about 8 ± 0 mm and 4 ± 0 mm, respectively. The negative control ethanol was found to have an inhibition halo of about 4 ± 2 mm. The root ethanol extract exhibited the greatest antimicrobial activity, with an inhibition zone of about 20 mm. The results of the tests are depicted in Table 8.

**Figure 4:**
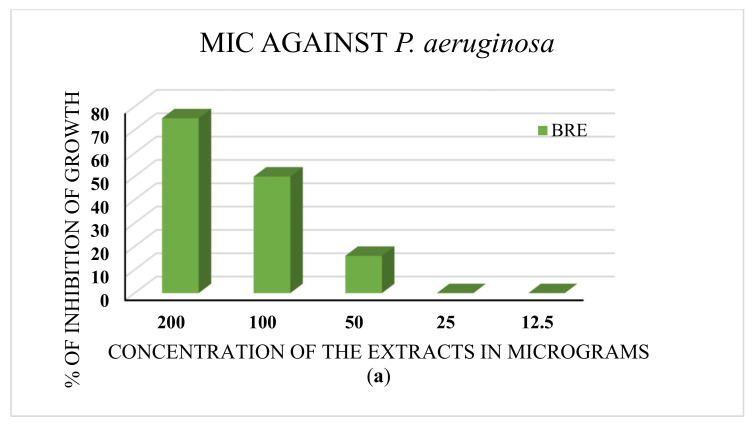

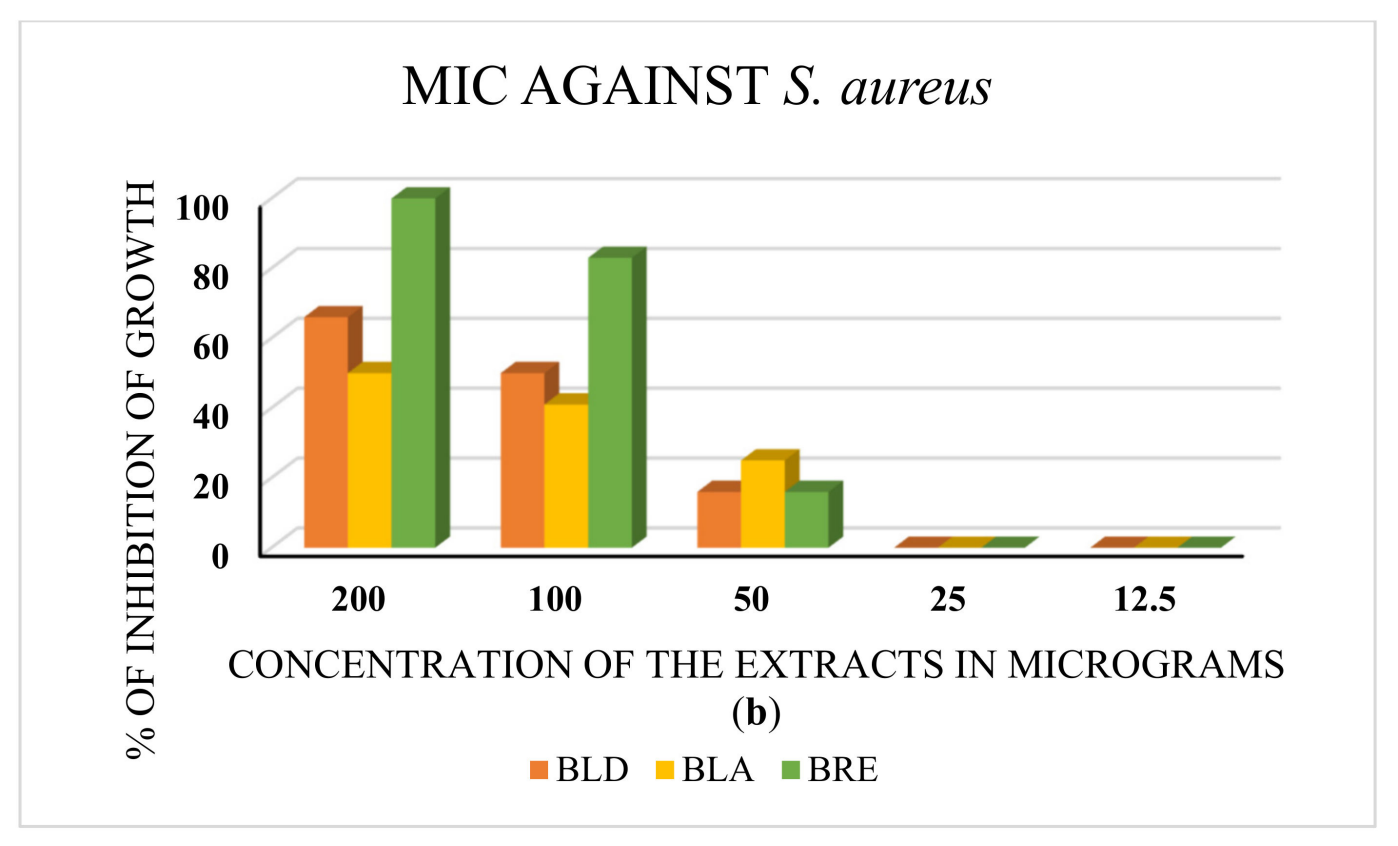
(**a**) The MIC test against *P. aeruginosa*. BRE is the root ethanolic extract of *B. diffusa*. The minimum concentration of the extract with antibacterial activity is 50 μg. (**b**) MIC test against *S. aureus.* BLD, BLA, and BRE denote the leaf decoction, the leaf aqueous extract, and the root ethanolic extract of *B. diffusa.* All extracts showed inhibition of bacterial growth at 50 μg concentration (MIC).

The minimum inhibitory concentration (MIC) was determined for the extracts that performed well in the disc diffusion assay. The ethanolic extract that showed inhibition against *P. aeruginosa* exhibited an MIC of 50 µg. The leaf decoction and the aqueous and ethanolic extracts of roots exhibited MICs of 50 µg against *S. aureus*. The results are depicted in Figure 4a,b.

### 2.5. GC–MS

**Figure 5:**
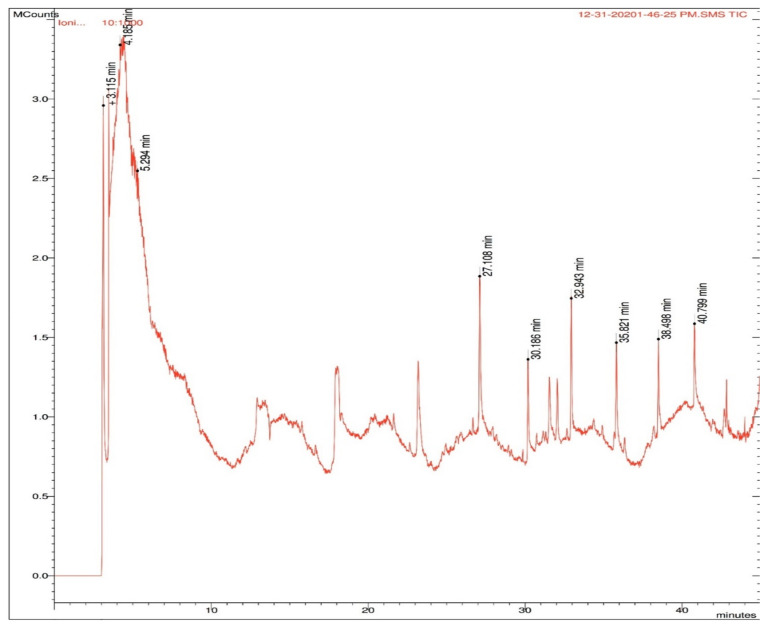
Peaks obtained in GC–MS analysis with retention times in *B. diffusa* root ethanolic extract. The compounds identified at the retention times are listed in Table 9.

The ethanolic extract of roots showed the maximum antimicrobial potential against both strains of bacteria. Since the ethanolic extract was prepared by cold extraction, the volatile compounds with antimicrobial activity were of interest; hence, the extract was subjected to GC–MS analysis. The peaks are shown in Figure 5, and the names of the compounds, along with their type, are mentioned in Table 9.

### 2.6. Molecular Interaction Findings

**Figure 6:**
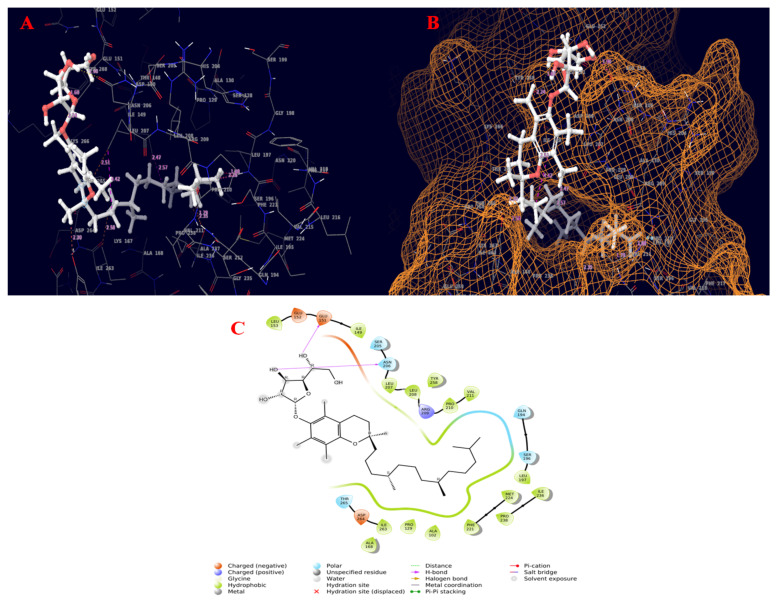
Molecular interaction diagram of quorum-sensing responsive protein complexed with 2-(1,2-dihydroxyethyl)-5-[[2,5,7,8-tetramethyl-2-(4,8,12-trimethyltridecyl)-3,4-dihydrochromen-6-yl]oxy]oxolane-3,4-diol. (**A**) Molecular interactions in 3D space; (**B**) ligand occupancies in binding pocket of target protein; (**C**) 2D ligand interactions, highlighting the hydroxyl group as key for hydrogen bond interactions with the target protein.

**Figure 7:**
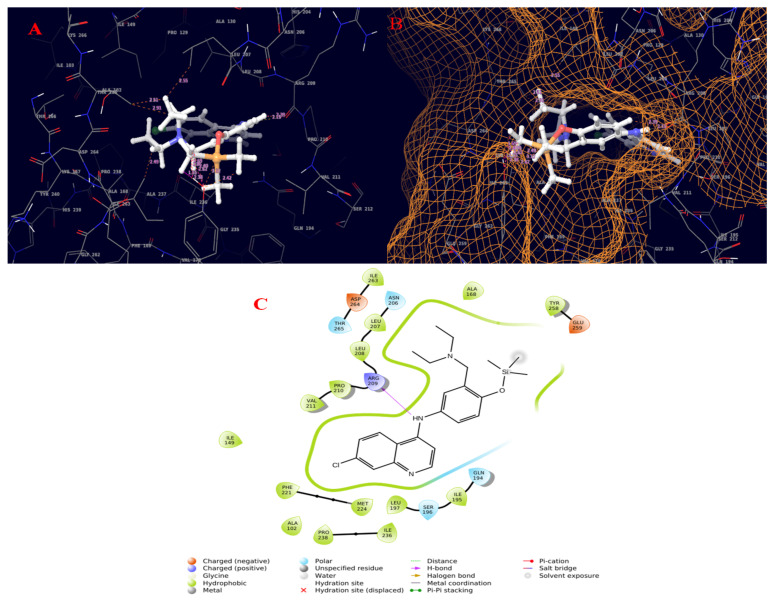
Molecular interaction diagram of quorum-sensing responsive protein complexed with amodiaquine (TMS derivative). (**A**) Molecular interactions in 3D space; (**B**) ligand occupancies in binding pocket of target protein; (**C**) 2D ligand interactions, highlighting the amide bond as key for interactions with the target protein.

**Figure 8:**
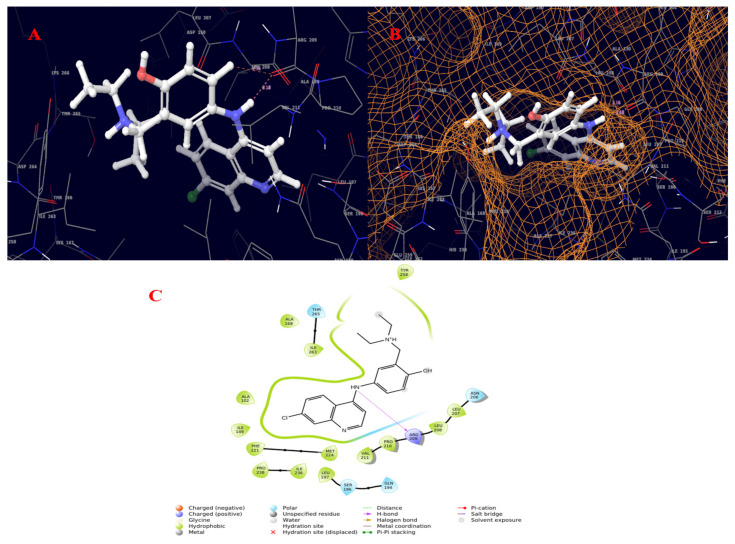
Molecular interaction diagram of quorum-sensing responsive protein complexed with amodiaquine. (**A**) Molecular interactions in 3D space; (**B**) ligand occupancies in binding pocket of target protein; (**C**) 2D ligand interactions, highlighting the amide bond as key for interactions with the target protein.

**Figure 9:**
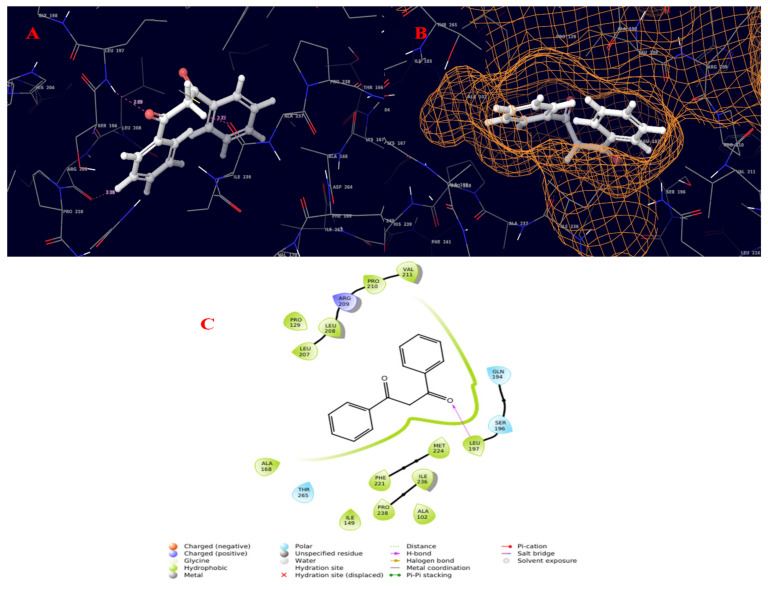
Molecular interaction diagram of quorum-sensing responsive protein complexed with 22-propen-1-one, 3-hydroxy-1,3-diphenyl-. (**A**) Molecular interactions in 3D space; (**B**) ligand occupancies in binding pocket of target protein; (**C**) 2D ligand interactions, highlighting the epoxy bond as key for interactions with the target protein.

The quorum-sensing regulator PqsR proteins from *Pseudomonas aeruginosa* were chosen as the molecular target for the current study. The chemical structures of the compounds identified by GC–MS were acquired from the NIST and PubChem databases. Molecular docking was conducted against 131 compounds utilizing GLIDE with the target protein, which generally prioritized results according to the program’s score calculation in the form of G-Score. The correctness of a docking pose is determined by the lowest-energy binding confirmation anticipated by the minimal scoring function. The G-score and GLIDE energy of the target proteins were all analyzed using the XP GLIDE docking technique. Table 10 depicts the interactions of amino acids with their bond lengths. According to the docking complex, the typical range of hydrogen bonds was roughly 3 Å. Our complex primary outcomes were below this range, indicating great interactions in the complexes. A total of 100 compounds successfully completed GLIDE docking, and the top-ranked results were highlighted. The ligand molecules with a cutoff GLIDE score below −7 are presented as 3D and 2D interaction diagrams (Figure 6, Figure 7, Figure 8 and Figure 9, Table 11).

## 3. Discussion

The current study qualitatively and quantitatively evaluated decoctions and maceration extracts (aqueous, ethanol, and chloroform) of *B. diffusa* leaves, stems, and roots, as well as conducted in vitro antioxidant and antimicrobial activity tests. The phytoconstituents present in the ethanolic extract of *B. diffusa* roots were also analyzed using in silico molecular docking. In the qualitative tests, the decoction of *B. diffusa* roots showed the presence of most phytochemicals. Proteins, flavonoids, carbohydrates, amino acids, and phenols showed a higher presence in contrast to saponins, quinone, and phytosterols. The *B. diffusa* leaf decoction showed a higher presence of proteins, terpenoids, carbohydrates, amino acids, and phenols in contrast to flavonoids and phytosterols. The *B. diffusa* stem decoction showed a higher presence of proteins, quinone, terpenoids, amino acids, and phenols in contrast to carbohydrates, saponins, flavonoids, and phytosterols. The *B. diffusa* leaf aqueous extract showed a higher presence of proteins, flavonoids, carbohydrates, amino acids, and phenols in contrast to saponins, terpenoids, and phytosterols. The *B. diffusa* stem aqueous extract showed a higher presence of proteins, quinone, carbohydrates, amino acids, and phytosterols in contrast to flavonoids and terpenoids. The *B. diffusa* root aqueous extract showed a higher presence of flavonoids, terpenoids, and carbohydrates in contrast to proteins, phenols, and phytosterols. Ethanol has a similar polarity to water; thus, its extracts also exhibited a higher presence of carbohydrates and amino acids than terpenoids and alkaloids. The stem ethanolic extract showed a lower presence of alkaloids in contrast to terpenoids, carbohydrates, and amino acids. The leaf ethanolic extract exhibited a higher presence of carbohydrates and amino acids than phenols and alkaloids. Chloroform is a less polar solvent; thus, its extracts revealed the presence of proteins in *B. diffusa* stems, leaves, and roots. Furthermore, the leaf chloroform extract showed the presence of phenols, the stem chloroform extract showed the presence of carbohydrates, phenols, and alkaloids, and the root chloroform extract showed the presence of terpenoids, carbohydrates, alkaloids, and phytosterols. These results agree with those obtained in [30] for the whole-plant *n*-hexane extract of *B. diffusa*. This *n*-hexane extract was prepared by hot extraction, similar to the decoction process in this study; therefore, it can be concluded that heating the plant material enriched it with most phytochemicals. The leaf and stem decoctions and aqueous extracts were similar with respect to the presence of compounds.

Certain observations could be made in the qualitative study, such as the presence of alkaloids only in the ethanolic extracts and chloroform extracts of stems and roots. Proteins were not present in any of the ethanolic extracts. Saponins and flavonoids were present only in the water-based extracts, except for the mild presence of flavonoids in the ethanolic extract of roots. Carbohydrates and phenols were present in all extracts of roots and leaves. Amino acids were unanimously absent in all chloroform extracts. The leaf chloroform extract was found to exhibit the lowest number of compounds. In the quantitative test of chlorophyll content, the leaves were high in comparison to stems, with a greater contribution of chlorophyll *a* (1.03 mg/g) to the total chlorophyll of 1.43 mg/g than chlorophyll *b* (0.4 mg/g). The chlorophyll content was measured in fresh leaves and stems, and the results were in line with those in [31], where the level of chlorophyll *a* was higher than that of chlorophyll *b*. The carbohydrate content was found to be 127 mg/g in *B. diffusa* root aqueous extract, 122 mg/g in root decoction, and 43.1 mg/g in root ethanolic extract, higher values than found in the extracts of leaves and stems. The lowest level of carbohydrates was found in the stem ethanol and chloroform extracts (2.96 mg/g and 2 mg/g, respectively). The water-based extracts and decoctions showed the maximum level of carbohydrates in each part. In the leaf, the maximum carbohydrate content was found in the aqueous extract that (42.1 mg/g) in contrast to the decoction (19.86 mg/g). In the stem, the maximum carbohydrate content was found in the decoction (41.39 mg/g) in contrast to the aqueous extract (36.3 mg/g). A similar result was found in cabbage, where the mid rib portion that is stalky had a higher carbohydrate content than the leaf blades according to [32]. In our study, the *B. diffusa* root was slight stalky in comparison to leaf and stem, which might have favored the presence of carbohydrates. In the protein estimation, the decoction of the *B. diffusa* leaves showed the highest concentration (305.8 mg/g), followed by the aqueous extract of the leaves. In the amino-acid estimation of *B. diffusa*, the decoction had the highest content (104 mg/g), followed by the stem ethanolic extract. Among the root extracts, the ethanolic extract showed the maximum value (48.8 mg/g). Similar findings were obtained for *Echinacae pallida*, which exhibited about 120 mg/g of amino-acid content [33].

The total phenolic content estimated by [30] was found to be highest in the ethyl acetate stem extract, in line with the highest value found in the stem decoction in this study (235 mg/g). A higher concentration of phenols was also found in the aqueous root extract (105 mg/g). Among the leaf extracts, the greatest phenolic content was found in the ethanolic extract (165 mg/g). The stem chloroform extract exhibited about 70 mg/g of phenolic content. In terms of the total phenolic content, there was a significant difference between the qualitative and quantitative tests, as the leaf ethanolic extract and stem chloroform extract showed a high level of phenolic content despite a mild color change in qualitative tests. The total flavonoid content was found to be high in *B. diffusa* decoctions of leaves (70 mg/g), stems (60 mg/g), and roots (60 mg/g). This result is supported by [34], where the flavonoid content was found to be highest in solvents of high polarity (methanol vs. ethyl acetate and petroleum ether). In the aqueous and ethanol extracts of roots, the flavonoid content was found to be 50 mg/g in each case. The stem aqueous and leaf aqueous extracts exhibited total flavonoid contents of 50 mg/g and 12 mg/g, respectively. Among the solvents used, water and ethanol were most polar, revealing good agreement between quantitative and quantitative tests of flavonoids. The decoctions of *B. diffusa* leaves and stems showed the maximum antioxidant potential in the DPPH assay, with the former exhibiting more than 60% inhibition with an IC_50_ value of about 136 µg at 200 µg concentration of the extract. The stem and root decoctions exhibited about 50% and 23% inhibition, respectively, at 200 µg concentration with IC_50_ values of 235 and 498 µg, respectively. Among the aqueous extracts, the stems showed the maximum inhibition of about 27.2%, followed by the leaves (22.2%) and roots (18.1%). Among the ethanolic extracts, the stems showed the maximum inhibition at 200 µg concentration (35.8%) with an IC_50_ value of about 273 µg. The leaf ethanolic extract exhibited about 27.2% inhibition, while the root ethanolic extract exhibited about 16.6% inhibition, corresponding to their IC_50_ values of about 369 and 635 µg, respectively. The root chloroform extract exhibited about 33.3% inhibition of DPPH, which was the highest among chloroform extracts, with an IC_50_ value of about 295 µg. The leaf chloroform extract exhibited about 20% inhibition, while the stem chloroform extract exhibited about 30.5% inhibition, with IC_50_ values of about 507 and 329 µg, respectively. Among the results obtained, the leaves and stems showed their maximum activity in decoctions, while the root showed its highest inhibitory potential in chloroform extracts. This result obtained may be due to the polarity of the water and the heat used when preparing the decoction; similarly, the methanol extract of *B. diffusa* with hot extraction exhibited maximum DPPH radical scavenging activity in comparison to the *n*-hexane and ethyl acetate extracts in [30]. The root chloroform extract was an exception to the polarity-related observations, exhibiting good activity.

The FRAP assay analysis revealed the maximum reducing potential of ferric ions in the aqueous extract of *B. diffusa* leaves, for which the optical density value was 0.14 when measured at 700 nm. The stem aqueous extract also showed a higher level of reducing potential (0.11 optical density) than the ethanolic extract (0.09 optical density), decoction (0.10 optical density), and chloroform extract (0.05 optical density). The maximum reducing activity of root was observed in its decoction. In [35], the reducing power of the *B. diffusa* leaf methanol extract was higher than that of the chloroform extract, and both extracts were prepared without the involvement of heat. Hence, the polarity of the solvent plays a greater role than heat when extracting the compounds required for the reducing power assay. The *B. diffusa* roots required heat for the solubilization of compounds responsible for reducing power in the decoction. In the ABTS assay, the aqueous extracts and decoctions of *B. diffusa* leaves, stems, and roots exhibited maximum inhibition, with the aqueous extracts revealing similar inhibition to ascorbic acid used as a standard (100% inhibition at 200 µg concentration). The stem aqueous extract exhibited 100% inhibition at 150 µg concentration. These results are in agreement with the study on *Hypericum cerastoids*, with 92% inhibition in contrast to the standard ascorbic acid that showed 96% inhibition. In the antimicrobial assay, the highest zone of inhibition against the Gram-negative bacterium *P. aeruginosa* was found in the decoctions of *B. diffusa* leaves and stems, as well as root ethanol extract, at 200 µg concentration, whose zones of inhibition were about 8 mm in diameter. The *B. diffusa* root ethanol extract had antibacterial activity at concentrations from 100 to 200 µg, with zones of inhibition of about 7 mm at 150 µg and 8 mm at 200 µg concentration. The *B. diffusa* root ethanolic extract stood out with a zone of inhibition even at the minimum concentration. In the case of *S. aureus*, the ethanolic extract was found to have the largest inhibition zone of about 20 mm at 200 µg. These results are in line with those in [30], where the methanolic extract was determined to have the highest antimicrobial activity. The MIC tests further confirmed 50 µg as the minimum concentration of extracts with an effect on bacterial growth in the disc diffusion assay.

The *B. diffusa* root ethanolic extract was subjected to GC–MS analysis, revealing alkaloids, phenols, flavonoids, and terpenoids. Since the ethanolic extract was prepared by cold extraction, the volatile compounds were retained in the extract. In order to investigate the volatile compounds, present in the extract, GC–MS analysis was applied. Amodiaquine is an alkaloid known for its antimalarial properties [36]. Dipyridamole is an anti-platelet agent found to exert antitumor activity [37]. 6-Aza-5,7,12,14-tetrathiapentacene is an alkaloid found in the GC–MS analysis of sesame oil and *Lilium candidum* flowers used to treat patients with chronic lower back pain [38]. Colchicines are antimitotic agents, with an effective role in protecting the liver from various hepatotoxic agents [39]. ⍺-Tocopherol was found to be effective as an antitumor agent against oral cancer [40]. Brousso-flavonol D is an anticancer agent related to pancreatic cancer [41]. 3,4-Dihydroxymandelic acid, ethyl ester, tri-TMS is a terpenoid found to be an antiangiogenic agent with anticancer activity in the methanol extract of *Rumex vasicarius* [42]. Phytochemicals are chemical compounds that help plants fight pathogens. *B. diffusa* has been examined extensively for its pharmacological qualities, such as anticancer, anti-inflammatory, and antioxidant properties. Many bioactive substances are found in *B. diffusa* [43].

The PqsR is a protein target that is an important intermediate in activating the PqsE protein responsible for the development, virulence, and biofilm production of bacteria [44]. Ligand-based drug design was recommended to address *P. aeruginosa* virulence in [45]. 2-Heptyl-4-hydroxyquinoline (HHQ) and the *Pseudomonas* quinolone signal (PQS) are the two factors which can induce the overexpression of PqsR via the PqsABCDE operon, thereby increasing the expression of PqsE [46]. Therefore, when a ligand can bind to the PqsR protein, it can inhibit the binding of either HHQ or PQS and can act as a potential quorum-sensing inhibitor. Applying the molecular docking approach to our selected pathogenic bacteria, the quorum-sensing responsive protein showed common interactions with the top-ranked molecules featuring TYR258, ARG209, ILE236, and LEU197. The molecular docking results revealed good binding affinity with the crude extract explored using in vitro assays, exhibiting superior antibacterial activity against the tested pathogen at increasing concentrations, according to the antimicrobial investigation. The whole plant of *B. diffusa* is used in traditional medicine to treat diabetes, stress, dyspepsia, abdominal discomfort, inflammation, jaundice, spleen enlargement, heart problems, bacterial infections, elephantiasis, night blindness, corneal ulcers, different hepatic illnesses, epilepsy, infertility, and menstrual pain, anas well as viral infections [30]. 2-(1,2-Dihydroxyethyl)-5-[[2,5,7,8-tetramethyl-2-(4,8,12-trimethyltridecyl)-3,4-dihydrochromen-6-yl]oxy]oxolane-3,4-diol also known as ⍺-tocopherol-β-d-mannoside was patented for the treatment of benign prostatic hypertrophy (BPH) and related aging symptoms using herbal extracts of *Ageratum* sp. [47]. Amodiaquine and its analogues were found to exhibit antimalarial activity with an IC_50_ value of 0.004 [48].

## 4. Materials and Methods

### 4.1. Plant Collection

*B. diffusa* plant samples were collected from the Bharathiar University Campus, Coimbatore, Tamil Nadu, India. The plants were collected in the months of September to November 2019. The leaves, stems, and roots were separated and allowed to dry in the shade for about 6 weeks. *B. diffusa* was identified by the Botanical Survey of India, Tamil Nadu Agricultural University, Coimbatore, Tamil Nadu, India, with certificate number is BSI/SRC/5/23/2019/Tech./293. The chemicals used in the current study were purchased from Himedia Laboratories Pvt. Ltd., Mumbai, India and Central Drug House Pvt Ltd., New Delhi, India through their dealers.

### 4.2. Extraction

*Maceration:* The shade-dried parts of *B. diffusa* were powdered, sieved, and then stored in an airtight container for further use. Fifty milligrams of the powdered leaves, stems, and roots of *B. diffusa* were dissolved in 50 mL of distilled water, ethanol, or chloroform in an airtight container for about 48 h with frequent agitation. After 48 h, the solvents were filtered using quantitative filter paper and stored for further use.

*Decoction:* Fifty milligrams of the powdered plant samples were dissolved in 50 mL distilled water, which was kept in a boiling water bath for about 15 min. The mixture was filtered using quantitative filter paper and stored for further use [49].

### 4.3. Qualitative Tests

- Carbohydrates

*Molisch’s test:* The extract was mixed with 2 mL of Molisch’s reagent, and the mixture was shaken properly. Then, 2 mL of concentrated H_2_SO_4_ was poured carefully along the side of the test tube, which was observed for the appearance of a violet ring at the interface [50].

- Proteins

*Xanthoproteic test:* One milliliter of concentrated nitric acid was added to 2–3 mL of test solution in a test tube. A positive test is indicated by the formation of a white precipitate, which upon heating turns yellow and finally dissolves, imparting to the solution a yellow color. The solution is cooled before carefully adding ammonium hydroxide or sodium hydroxide in excess, whereby the yellow solution deepens into an orange color [40].

- Amino Acids

*Ninhydrin test:* Five drops of 0.2% ninhydrin solution in acetone were added to 1 mL of amino-acid solution. The mixture was boiled over a water bath for 2 min and allowed to cool, before observing the formation of a blue color [50].

- Flavonoids

*Alkaline reagent test:* Two milliliters of 2.0% NaOH mixture was mixed with aqueous plant crude extract; a concentrated yellow color was produced, which became colorless upon adding two drops of diluted acid to the mixture. This result showed the presence of flavonoids [51].

- Phenols

*Lead acetate test:* To 0.2 mL of extract, 2 mL of aqueous sodium carbonate was added, followed by the addition of 0.2 mL of Folin’s reagent. A color change to blue or gray indicated the presence of phenols [52].

- Alkaloids

*Test for alkaloids:* To 2 mL of extract, 2 mL of concentrated HCl was added. Then, a few drops of Mayer’s reagent were added. The presence of green color, white precipitate, or turbidity indicated the presence of alkaloids [53].

- Phytosterols

*Salkowski’s test:* The chloroform extract was treated with concentrated H_2_SO_4_ and observed for the formation of a red color [51].

- Saponins

*Foam test:* A fraction of the extract was vigorously shaken with 20 mL of water in a graduated cylinder for 15 min, which was observed for the presence of persistent foam [51].

- Terpenoids

*Salkowski’s test:* About 5.0 mL of extract was mixed with 2.0 mL of chloroform and 3.0 mL of concentrated H_2_SO_4_**.** A reddish-brown color at the interface indicated the presence of terpenoids [51].

- Quinone

*HCl method:* To 1.0 mL of extract, a few drops of concentrated HCl was added. A yellowish-brown color indicated the presence of quinone [40,41].

### 4.4. Quantitative Tests

- Chlorophyll

Chlorophyll was extracted from 1 g of the sample using 20 mL of 80% acetone. The supernatant was transferred to a volumetric flask after centrifugation at 5000 rpm for 5 min. The extraction was repeated until the residue became colorless. The volume in the flask was made up to 100 mL with 80% acetone. The absorbance of the extract was read in a spectrophotometer at 645 nm and 663 nm against an 80% acetone blank. The amount of total chlorophyll in the sample was calculated using the following formula: V total chlorophyll = 20.2 (A645) + 8.02 (A663) × V/1000 × W, where, V is the final volume of the extract, and W is the fresh weight of the leaves.

The amounts of chlorophyll *a* and *b* can be calculated as follows: 12.21 (A633) − 2.81 (A645) × V/1000 × W and 20.13 (A645) − 5.03 (A663) × V/1000 × W, respectively. The results are expressed as mg chlorophyll/g sample [54].

- Protein

The protein estimation was conducted using bovine serum albumin as a standard [55]. The plant extracts were taken in a concentration within the working standards, i.e., 40–200 µg. All tubes were made to 1.0 mL using distilled water, while 1.0 mL of distilled water served as a blank. Next, 5 mL of alkaline reagent was added to all tubes before incubating for 10 min at 37 °C. Then, 0.2 mL of Folin’s phenol reagent was added before incubating for 30 min. The intensity of the color developed was measured at 660 nm against the blank.

- Carbohydrate

Glucose was used as a standard for the carbohydrate analysis. The working standard was prepared at a concentration of 100 µg/mL. About 0.2 to 1.0 mL of the working standards were taken in test tubes, while 1.0 mL water served as a blank. The volume was made up to 1 mL in all tubes with distilled water along with the sample tubes, with a concentration range within the working standards. Then, 4 mL of anthrone reagent was added before heating for 8 min in a boiling water bath. The mixture was cooled rapidly and observed for the formation of a green to dark-green color at 630 nm [56].

- Amino acids

The amino-acid estimation was achieved using leucine as a standard [57]. Working standards within a concentration range of 20–100 µg were taken. Samples of plant extracts with concentration within the working standards were also taken, and all tubes were made to 1.0 mL along with the blank and standard. About 1.0 mL of ninhydrin reagent (0.8 g stannous chloride in 500 mL of 0.2 M citrate buffer, pH 5.0, added to 20 g of ninhydrin in 500 mL of acetone) was added to all tests tubes, which were left in a boiling water bath for about 20 min. About 5.0 mL of diluent made with an equal volume of propanol and water was added to all tubes before incubating for 15 min. The development of a purple color was read at 570 nm in a spectrophotometer.

- Flavonoids

About 1 mL of the test sample and 4 mL of water were added to a volumetric flask (10 mL volume). Then, 0.3 mL of 5% sodium nitrite and 0.3 mL of 10% aluminum chloride were added after 5 min. After 6 min of incubation at room temperature, 1 mL of 1 M sodium hydroxide was added to the reaction mixture. The final volume was immediately made up to 10 mL with distilled water. The absorbance of the sample was measured against the blank at 510 nm [53].

- Phenols

The phenol estimation was conducted using gallic acid as the standard. To varying concentrations of the standard and extracts, about 0.5 mL of Folin–Ciocâlteu reagent was added, and the mixture was incubated for 3 min. About 2.0 mL of sodium carbonate 20% was added, and the mixture was allowed to stand in the dark for 1 h. The blue color developed was measured at 650 nm [58].

### 4.5. In Vitro Antioxidant Assays

- DPPH Scavenging Assay

The DPPH assay was conducted using *B. diffusa* extracts of varying concentrations (50, 100, 150, and 200 μg) along with ascorbic acid standard at the same concentrations. About 3.0 mL of 0.1 mM DPPH dissolved in methanol was added to all tubes. The tube without any extract and 3.0 mL of DPPH served as the A_0_, while methanol was used as the blank. The mixture was allowed to react at room temperature for 30 min in the dark. After 30 min of incubation, the discoloration of the purple color was measured at 518 nm in a spectrophotometer [59]. The radical-scavenging activity was calculated as follows:

$$\%\;\mathrm{scavenged}\;\mathrm{DPPH}\;=\;\frac{A_{0}-A_{1}\times 100}{A_{0}},$$

where A_0_ is the absorbance of the control, and A_1_ is the absorbance in the presence of various extracts.

- FRAP Assay

Different concentrations of extracts of *B. diffusa* (125, 250, 500, 750, and l000 μg) were made up to 1 mL with distilled water, and a tube with 1 mL of distilled water alone was used as the blank. About 2.5 mL of phosphate buffer (0.2 M, pH 6.6) and 2.5 mL of potassium ferricyanide (1%) were added to all extracts. The mixture was incubated at 50 °C for 20 min. A portion (2.5 mL) of trichloroacetic acid (10%) was added to the mixture, which was then centrifuged for 10 min at 1000× *g.* Then, 2.5 mL of the upper layer of the solution was mixed with 2.5 mL of distilled water and 0.5 mL of FeCl_3_ (0.1%), and the absorbance was measured at 700 nm in a spectrophotometer. A higher absorbance of the reaction mixture indicated a greater reductive potential of the extracts [60].

- ABTS Assay

The ABTS assay was performed using a diluted stock solution prepared by mixing an equal volume of 7 mM ABTS in phosphate-buffered saline and 2.4 mM potassium persulfate solution. The stock was left to incubate in the dark for about 14 h. The stock was diluted with the help of methanol or ethanol until the OD value reached 0.7 units at 734 nm. The plant extracts ranging in concentration from 50–200 µg were added to the test tubes and mixed with 1.0 mL of diluted ABTS solution. The mixture was left to incubate in the dark for about 7 min, and then the OD values were measured using a spectrophotometer. About 1.0 mL of the diluted ABTS solution was also incubated in a separate tube in the same manner, which served as the control. Ethanol or methanol was used as the blank. The percentage inhibition of ABTS was calculated as follows: (A*c* − A*s*/A*c*) × 100, where A*c* is the OD of the control, and A*s* is the OD of the samples [61].

### 4.6. Antimicrobial Assay

- Agar disc diffusion method

The antimicrobial assay [62,63] was performed against the Gram-negative bacterium *P. aeruginosa* (ATCC 27853) and Gram-positive bacterium *S. aureus* (ATCC 25923) using the decoctions and aqueous, ethanol, and chloroform extracts of *B. diffusa* leaves, stems, and roots at concentrations of 100, 150, and 200 µg. The colony suspensions of the bacterial strain were maintained according to the 0.5 McFarland standard. Distilled water, ethanol, and chloroform were used as the negative controls, and a ceftazidime antimicrobial disc was used as the positive control for *P. aeruginosa*, while a vancomycin antimicrobial disc was used as the positive control for *S. aureus.* The medium used was Mueller–Hinton Agar. About 100 µL of microbial culture was spread all over the plate containing the medium. The discs immersed in the extracts of various concentrations were immediately placed on the plates after spreading the microbial culture. The plates were then incubated in an inverted position for about 16–18 and 24 h at 37 °C. After incubation, the plates were observed for the zone of inhibition, and its diameter was measured in mm. The antimicrobial assay followed the CLSI criteria.

An MIC test was performed in order to find the minimum concentration of the extracts with antibacterial activity, which performed well in the disc diffusion assay. The MIC using the broth dilution method was determined as a function of the turbidity measured in a spectrometer at 625 nm. The inhibition of bacterial growth was calculated as follows:

OD of positive control − OD of the sample/value of positive control − negative control × 100.

### 4.7. Computational Validation

- **Molecular docking**

All docking calculations were performed using the GLIDE program’s extra precision (XP) mode in the Maestro Platform of Schrodinger software. The docked ligand and protein interaction was assessed for optimum conformation using the best pose displayed in a pose viewer. The ligand interaction module was used to obtain the 2D interactions of protein and ligand (Schrödinger Release 2021-3: Glide, Schrödinger, LLC, New York, NY, USA, 2021). The three-dimensional structure with amino-acid interactions and active site cavity was analyzed in Maestro.

- **Structure elucidation of small molecules**

The metabolic compounds extracted from *B. diffusa* were chosen for this study; the molecular structures of the compounds were determined using GC–MS. Overall, 139 molecules were identified, the three-dimensional structures of which were retrieved from the NIST and PubChem databases in 2D and 3D SDF (Structure Data File) and Mol format (Supplementary Table S1). Using the Maestro platform with the LigPrep module, the molecular structures were computationally generated as 32 stereoisomers using OPLS4 force field energy minimization. Tautomers were created, and conformations of the ligand’s orientation were investigated (Schrödinger Release 2021-3:LigPrep, Schrödinger, LLC, New York, NY, USA, 2021).

- **Target preprocessing**

The quorum-sensing response, which involves three regulons, Las, Rhl, and Pqs, governs the expression of numerous virulence factors, and LasR and RhlR are transcriptional regulators of the Las and Rhl systems [48]. Accordingly, we chose the quorum-sensing responsive protein (PDB ID: 4JVC) as the target. The bond orders were assigned to the protein structure, hydrogens were added, zero-order bonds to metals and disulfide bonds were created, selenomethionines were converted to methionines, and missing side-chains were filled using Prime; the inhibitor present in the crystallized protein was removed, and the H-bonds were optimized, before final energy minimization using the OPLS4 force field.

- **Statistical analysis**

The values in the tables are expressed as the mean ± the standard error (*n =* 3). The IC_50_ values in the DPPH assay were calculated using the trend lines for each extract (*y* = *ax* + *b*), where *y* was considered to be 50, *a* and *b* were obtained from the graph, and *x* was the IC_50_ value obtained from the equation.

## 5. Conclusions

In the present study, hot extraction allowed for high-quality decoctions of *B. diffusa* leaves, stems, and root, whereas the use of organic solvents such as ethanol with higher polarity aided in the solubility of most alkaloids in comparison to flavonoids and phenols. The top-ranked molecules in *B. diffusa* extract were identified as 2-(1,2-dihydroxyethyl)-5-[[2,5,7,8-tetramethyl-2-(4,8,12-trimethyltridecyl)-3,4-dihydrochromen-6-yl]oxy]oxolane-3,4-diol, amodiaquine TMS derivative, amodiaquine, and 2-propen-1-one, 3-hydroxy-1,3-diphenyl, which were subsequently evaluated using GLIDE docking. The efficacy of these potential drug candidates was also evaluated through in silico molecular docking, which revealed the key molecular interactions, particularly hydrogen bond contacts. The present study revealed the difference between hot and cold extraction procedures, as well as the impact of solvent polarity, when extracting different compounds from *B. diffusa*, along with their antimicrobial activity against *P. aeruginosa* and *S. aureus* (infectious pathogens to humans). As potential candidates against *Pseudomonas* infection, our top-ranked molecules are expected to be carried over into clinical phase trials; however, the presented drugs require further confirmation using an in vivo model.

## Figures and Tables

**Table 1:** Qualitative tests using different extracts of *B. diffusa* leaves, stems, and roots. LA, LD, LC, and LE represent the aqueous extract, decoction, chloroform extract, and ethanol extract of *B. diffusa* leaves. SA, SD, SCm and SE represent the aqueous extract, decoction, chloroform extract, and ethanol extract of stems. RA, RD, RC, and RE represent the aqueous extract, decoction, chloroform extract, and ethanol extract of *B. diffusa* roots. + indicates the presence of a particular compound, while ++ indicates a higher presence, according to a rapid and intense color change; –− indicates the absence of a particular compound.

S.NO	TESTS	LA	LD	LC	LE	SA	SD	SC	SE	RA	RD	RC	RE
1.	Protein	++	++	++	−	++	++	++	−	+	++	++	−
2.	Saponins	+	−	−	−	−	+	−	−	−	+	−	−
3.	Flavonoids	++	+	−	−	+	+	−	−	++	++	−	+
4.	Quinone	−	−	−	−	++	++	−	−	+	+	−	−
5.	Terpenoids	+	++	−	−	+	++	−	++	++	++	++	+
6.	Carbohydrates	++	++	−	++	++	+	++	++	++	++	++	++
7.	Aminoacids	++	++	−	++	++	++	−	++	−	++	−	++
8.	Phenols	++	++	+	+	++	++	+	−	+	++	−	−
9.	Alkaloids	−	−	−	+	−	−	+	+	−	−	+	+
10.	Phytosterols	+	+	−	−	++	+	−	−	+	+	+	−

**Table 2:** Chlorophyll content present in *B. diffusa* leaves and stems.

S. No	*B. diffusa* Parts	Total Chlorophyll Content in mg/g (Mean ± SE)	Chlorophyll *a* Content in mg/g (Mean ± SE)	Chlorophyll *b* Content in mg/g (Mean ± SE)
1.	Leaves	1.43 ± 0.05	1.03 ± 0.05	0.4 ± 0.05
2.	Stem	0.35 ± 0.02	0.20 ± 0.02	0.15 ± 0.02

**Table 3:** Carbohydrate content present in *B. diffusa* leaves, stems, and roots.

S. No	Extracts	*B. diffusa* Leaf Carbohydrate in mg/g (Mean ± SE)	*B. diffusa* Stem Carbohydrate in mg/g (Mean ± SE)	*B. diffusa* Root Carbohydrate in mg/g (Mean ± SE)
1.	Decoction	19.86 ± 1.14	41.39 ± 1.6	122.0 ± 1.0
2.	Aqueous	42.1 ± 2.9	36.3 ± 3.47	127.16 ± 5.30
3.	Ethanol	7.97 ± 0.21	2.96 ± 0.50	43.1 ± 0.1
4.	Chloroform	-	2.00 ± 0.05	20.56 ± 0.2

**Table 4:** Protein content present in *B. diffusa* leaves, stems, and roots.

S. No	Extracts	*B. diffusa* Leaf Protein in mg/g (Mean ± SE)	*B. diffusa* Stem Protein in mg/g (Mean ± SE)	*B. diffusa* Root Protein in mg/g (Mean ± SE)
1.	Decoction	305.8 ± 5.8	75 ± 0	30.8 ± 0.8
2.	Aqueous	98.8 ± 0.4	74.2 ± 0.68	25 ± 0
3.	Ethanol	30 ± 0	26.2 ± 1.6	25 ± 0
4.	Chloroform	23.8 ± 3.8	42.7 ± 3	60.5 ± 1.6

**Table 5:** Amino-acid content present in *B. diffusa* leaves, stems, and roots.

S. No	Extracts	*B. diffusa* Leaf Amino Acids in mg/g (Mean ± SE)	*B. diffusa* Stem Amino Acids in mg/g (Mean ± SE)	*B. diffusa* Root Amino Acids in mg/g (Mean ± SE)
1.	Decoction	104.4 ± 2.2	18.8 ± 0.5	23.3 ± 0.9
2.	Aqueous	11.1 ± 1.4	15 ± 2.5	18 ± 4.9
3.	Ethanol	87.2 ± 1.4	101.6 ± 0.9	48.8 ± 1.1
4.	Chloroform	11.6 ± 2.0	6.1 ± 0.5	18.8 ± 1.1

**Table 6:** Flavonoid content present in *B. diffusa* leaves, stems, and roots.

S. No	Extracts	*B. diffusa* Leaf Flavonoids in mg/g (Mean ± SE)	*B. diffusa* Stem Flavonoids in mg/g (Mean ± SE)	*B. diffusa* Root Flavonoids in mg/g (Mean ± SE)
1.	Decoction	70 ± 0.01	60 ± 0.00	60 ± 0.00
2.	Aqueous	12 ± 0.03	50 ± 0.01	50 ± 0.01
3.	Ethanol	-	-	50 ± 0.01
4.	Chloroform	-	-	-

**Table 7:** Phenolic content present in *B. diffusa* leaves, stems, and roots.

S. No	Extracts	*B. diffusa* Leaf Phenols in mg/g (Mean ± SE)	*B. diffusa* Stem Phenols in mg/g (Mean ± SE)	*B. diffusa* Root Phenols in mg/g (Mean ± SE)
1.	Decoction	36 ± 0.20	235 ± 0.40	14 ± 0.2
2.	Aqueous	37 ± 0.30	15 ± 0.45	105 ± 0.57
3.	Ethanol	165 ± 0.25	-	-
4.	Chloroform	-	70 ± 0.01	-

**Table 8:** Zone of inhibition of different extracts of *B. diffusa* leaves, stems, and roots against *P. aeruginosa* and *S. aureus*.

Zone of Inhibition against *P. aeruginosa.*										
S. No	Extracts	*B. diffusa* Leaf Diameter of Zone of Inhibition in mm Mean ± SE			*B. diffusa* Stem Diameter of Zone of Inhibition in mm Mean ± SE			*B. diffusa* Root Diameter of Zone of Inhibition in mm Mean ± SE		
		100 µg	150 µg	200 µg	100 µg	150 µg	200 µg	100 µg	150 µg	200 µg
1.	Decoction	-	-	8 ± 1	-	-	8 ± 2	-	-	7 ± 1
2.	Aqueous	-	-	-	-	-	-	-	-	-
3.	Ethanol	-	-	-	-	-	-	-	7 ± 0	8 ± 0
4.	Chloroform	-	-	-	-	-	-	-	-	-
5.	Negative control (NC)	-								
6.	Positive control (PC)	18 ± 2								
Zone of inhibition against *S. aureus.*										
1.	Decoction	5 ± 1	7 ± 1	8 ± 1	-	4 ± 0	6 ± 2	-	-	10 ± 0
2.	Aqueous	6 ± 0	8 ± 0	9 ± 0	-	4 ± 0	6 ± 0	-	-	9 ± 2
3.	Chloroform	-	-	8 ± 0	-	-	-	-	4 ± 1	4 ± 0
4.	Ethanol	-	-	17 ± 2	-	5 ± 0	8 ± 1	-	6 ± 0	20 ± 2
5.	Negative control (NC)	4 ± 2								
6.	Positive control (PC)	20 ± 1								

**Table 9:** Different compounds obtained from GC–MS along with compound type and retention time.

S. No.	Compounds	Retention Time (min)	% of Total	Compound Type	Type
1.	Phthalic acid, monoamide, *N*,*N*-diheptyl, pentyl ester	27,108	15.571	Alkaloid	Antimicrobial [22]
2.	Xanthine, 8-[3-iodocyclopentyl]-1,3-dipropyl-	27,108	15.571	Alkaloid	-
3.	Benzoxazole, 2,2’-(2,5-thiophenediyl)bis[5 -(1,1-dimethylethyl)-	27,108	15.571	Alkaloid	-
4.	Benzamide, *N*,*N*-didecyl-4-methoxy-	27,108	15.571	Alkaloid	-
5.	14-Acetyldictyocarpine	30,186	6.952	Alkaloid	-
6.	2-(2-Hydroxy-4-octyloxyphenyl)-5-(4-octyloxyphenyl)pyrimidine	30,186	6.952	Alkaloid	-
7.	Silanamine, *N*-[(17β)-3,17-bis[(trimethylsilyl)oxy]estra-1,3,5(10)-trien-2-yl]-1,1,1-trimethyl-	30,186	6.952	Alkaloid	
8.	Vobtusine, anhydrode(methoxycarbonyl)-	30,186	6.952	Alkaloid	
9.	Dipyridamole	30,186	6.952	Alkaloid	Antimicrobial [23]
10.	*trans*-4-Nitro-4’-(octadecyloxy)chalcone	30,186	6.952	Alkaloid	Antimicrobial [24]
11.	Terbutaline, *N*-trifluoroacetyl-o,o,o-tris(trimethylsilyl)deriv.	32,943	10.516	Alkaloid	
12.	1,6-bis(4’-Chlorophenyl)-3-methyl-4,5-dihydropyrazolo[3,4-b]pyridine	32,943	10.516	Alkaloid	
13.	6-Aza-5,7,12,14-tetrathiapentacene	32,943	10.516	Alkaloid	
14.	Benzeneethanamine, *N*-[(pentafluorophenyl)methylene]-.beta.,3,4-tris[(trimethylsilyl)oxy]-	32,943	10.516	Alkaloid	
15.	Piperazine-1-ethanol, 4-(2-diethylaminosulfonyll-4-nitrophenyl)-	32,943	10.516	Alkaloid	
16.	*O*,*O*,*O*-Tris-trimethylsilyl-epinephrine	32,943	10.516	Alkaloid	
17.	Amodiaquine	32,943	10.516	Alkaloid	Antimicrobial [25]
18.	1,6-bis(4’-Chlorophenyl)-3-methylpyrazolo[3,4-b]pyridine	32,943	10.516	Alkaloid	
19.	Amodiaquine TMS derivative	32,943	10.516	Alkaloid	Antimicrobial [25]
20.	Cobalt, bis(.eta.-5-piperidinylcyclopentadienyl)-	32,943	10.516	Alkaloid	Antimicrobial [26]
21.	Glycine, *N*-methylsulfonyl-*N*-(4-chloro-2-methylphenyl)-, 4-benzylpiperidide	32,943	10.516	Alkaloid	-
22.	*N*-(4-{1-[4-(4-Acetylaminophenoxy)-3-methoxyphenyl]-2-[(4-acetylamino-phenyl)methylamino]ethoxy}phenyl)acetamide	38,499	6.315	Alkaloid	-
23.	Cholest-2-eno[2,3-b]indole, 1′-methyl-5’-methoxy-	38,499	6.315	Alkaloid	-
24.	4-(4-Ethoxycarbonylbuta-1,3-dienyl)-1-methyl-2,5-diphenyl-1H-pyrrole-3-carboxylic acid, ethyl ester	38,499	6.315	Alkaloid	-
25.	Cholest-2-eno[2,3-b]indole, 1′-methyl-4’-methoxy-	38,499	6.315	Alkaloid	-
26.	(3,4-Dimethyl-5-oxo-2,5-dihydro-1*H*-pyrrol-2-yl)-[4,4-dimethyl-5-(2,3,3-trimethyl	38,499	6.315	Alkaloid	-
27.	2,16,27,28-Tetraazaheptacyclo[15.7.1.1(3,25).1(5,8).1(10,13).1(15,19).0(18,21)]nonacosa-1,3,5,7,9,11,13(28),14,17,19(29)-decaene-20,22-dione,12-ethyl-21-methoxy-6,11,26,29-tetramethyl-7-ethenyl-	38,499	6.315	Alkaloid	-
28.	7-Chloro-1-[[3-[dimethylamino]propyl]imino-2-ethyl-1,3,4,10-tetrahydro-3-(*p*-trifluorophenyl)-9(2*H*)acridinone	38,499	6.315	Alkaloid	-
29.	(3,4-Dimethyl-5-oxo-2,5-dihydro-1*H*-pyrrol-2-yl)-[4,4-dimethyl-5-(2,3,3-trimethyl-5-methylthio-3,4-dihydro-2*H*-pyrrol-2-ylmethylene)pyrrolidin-2-ylene]-thioacetic acid, S-(*tert*-butyl) ester	38,499	6.315	Alkaloid	-
30.	Silanamine, *N*-[(17β)-3,17-bis[(trimethylsilyl)oxy]estra-1,3,5(10)-trien-2-yl]-1,1,1-trimethyl-	38,499	6.315	Alkaloid	-
31.	*N*-[3-(4-Fluoro-phenoxy)-5-nitro-phenyl]-2-(4-trifluoromethyl-5,6-dihydro-benzo[h]quinazolin-2-ylsulfanyl)-acetamide	38,499	6.315	Alkaloid	-
32.	Cholest-2-eno[2,3-b]indole, 1′-methyl-7’-methoxy-	38,499	6.315	Alkaloid	-
33.	Fluvalinate	38,499	6.315	Alkaloid	-
34.	⍺-Lumicolchicine	40,979	7.639	Alkaloid	-
35.	β-Lumicolchicine	40,979	7.639	Alkaloid	-
36.	γ-Lumicolchicine	40,979	7.639	Alkaloid	-
37.	1*H*-Pyrrolo[2,3-f]quinoline-2,7,9-tricarboxylic acid, 2-ethyl 7,9-dimethyl ester	40,979	7.639	Alkaloid	-
38.	6,6’-Bis(phenylethynyl)-2,2’-bipyridine	40,979	7.639	Alkaloid	-
39.	dl-⍺-Tocopherol succinate	27,108	15.571	Flavonoid	-
40.	⍺-Tocopherol-β-d-mannoside	27,108	15.571	Flavonoid	Antimicrobial [27]
41.	Vitamin E acetate	27,108	15.571	Flavonoid	Antimicrobial [28]
42.	Brousso-flavonol D	30,186	6.952	Flavonoid	-
43.	⍺-Tocopherol	27,108	15.571	Phenol	Antimicrobial [27]
44.	Eupomatilone-3	27,108	15.571	Lignan	-
45.	Eupomatilone-4	27,108	15.571	Lignan	-
46.	Irieol	27,108	15.571	Terpenoid	-
47.	9-Deacetoxy-14,15-deepoxyxeniculin	30,186	6.952	Terpenoid	-
48.	Benzeneacetic acid, alpha.,3,4-tris[(trimethylsilyl)oxy]-, trimethylsilyl ester	32,943	10.516	Terpenoid	-
49.	Benzoic acid, 2,4-bis[(trimethylsilyl)oxy]-, trimethylsilyl ester	32,943	10.516	Terpenoid	-
50.	Silane, [[4-[1,2-bis[(trimethylsilyl)oxy]ethyl]-1,2-phenylene]bis(oxy)]bis[trimethyl-	32,943	10.516	Terpenoid	-
51.	Benzeneacetic acid, alpha.,3,4-tris[(trimethylsilyl)oxy]-, methyl ester	32,943	10.516	Terpenoid	-
52.	Benzoic acid, 2,5-bis(trimethylsiloxy)-, trimethylsilyl ester	32,943	10.516	Terpenoid	-
53.	3,4-Dihydroxymandelic acid, ethyl ester, tri-TMS	32,943	10.516	Terpenoid	-
54.	Benzoic acid, 2,3-bis[(trimethylsilyl)oxy]-, trimethylsilyl ester	32,943	10.516	Terpenoid	-
55.	3,5-Dihydroxybenzoic acid 3TMS	32,943	10.516	Terpenoid	-
56.	Isoproterenol tri-TMS derivative	32,943	10.516	Terpenoid	-
57.	*N*-(Trifluoroacetyl)-*O*,*O*′,*O*″-tris(trimethylsilyl)norepinephrine	32,943	10.516	Terpenoid	-
58.	2,6-Dihydroxybenzoic acid 3TMS	32,943	10.516	Terpenoid	Antimicrobial [29]
59.	*trans*,*trans*-1,1′-(*m*-Phenylene)bis(3-(*p*-(methylthio)phenyl)-2-propen-1-one)	35,821	9.054	Terpenoid	-
60.	5⍺-Cholestan-19-oic acid, 2β-methoxy-(CAS)	38,499	6.315	Terpenoid	-
61.	Estra-1,3,5(10)-triene-7,17-dione, 3-[(trimethylsilyl)oxy]-	40,979	7.639	Terpenoid	-
62.	5,12-d-Ethano(furo[2,3,4-mn]oxepino[2,3,4-ed]anthracen-9-ol-2-one), 6-methyl-12acetoxy-2a,3,4,4a,5,7,8a-octahydro-	40,979	7.639	Terpenoid	-
63.	Kanzonol M; CHEBI:171678; 7-hydroxy-3-[2-hydroxy-4-methoxy-3-(3-methylbut-2-enyl)phenyl]-5-methoxy-3,4-dihydro-2*H*-chromene-8-carbaldehyde	40,979	7.639	Terpenoid	-
64.	Oxirane	3115	7.639	-	-

**Table 10:** Compounds obtained from crude extract of *B. diffusa* profiled from GC–MS exploited against the pathogenic bacterial quorum-sensing protein using molecular docking experiment. Complex interaction molecules and their minimum binding scores are listed; ARG209, TYR258, ILE236, and LEU197 interactions were conserved among the top four ranked molecules.

PubChem ID	Molecule Name	Amino-Acid Interaction	Bond Length	Glide Score	Glide Energy
597057	2-(1,2-Dihydroxyethyl)-5-[[2,5,7,8-tetramethyl-2-(4,8,12-trimethyltridecyl)-3,4-dihydrochromen-6-yl]oxy]oxolane-3,4-diol	ILE149, GLU151, LEU207, TYR258, ILE263, ILE236, GLN194, LEU197, ASN206	(2.47, 2.57), (2.24, 1.60), (2.51), 2.30, 2.58, 2.35, 1.79, (1.48, 2.26), 1.90	−8.264	−47.143
632012	Amodiaquine TMS derivative	TYR258, ARG209, ILE263, THR265, LEU207	(2.38, 1.55, 1.86, 1.69, 2.40, 3.12, 2.42), (2.15, 2.39), 2.49, (2.51, 2.91), 2.55	−8.131	−38.567
2165	Amodiaquine	ARG209	2.18, 2.16	−7.919	−42.785
641324	2-Propen-1-one, 3-hydroxy-1,3-diphenyl-	ILE236, LEU197, ARG209	2.22, 2.09, 2.20	−7	−32.675

**Table 11:** Compounds obtained from crude extract of *B. diffusa* profiled from GC–MS exploited against the pathogenic bacterial quorum-sensing protein using molecular docking experiment. Complex interaction molecules with minimum binding scores below the cutoff value of −5 are listed.

PubChem ID	Compound Name	Glide Score	Glide Energy
597057	⍺-Tocopherol-β-d-mannoside	−8.264	−47.143
632012	Amodiaquine TMS derivative	−8.131	−38.567
2165	Amodiaquine	−7.919	−42.785
641324	2-Propen-1-one, 3-hydroxy-1,3-diphenyl-	−7	−32.675
5363841	3-Benzyl-4-hydroxy-3-pentene-2-one	−6.75	−22.638
136819	1,2,3-Triphenylazulene	−6.532	−20.565
457194	⍺-Conidendrin	−6.406	−27.392
103763	2*H*-1-Benzopyran-6-ol, 3,4-dihydro-2-methoxy-2,5,7,8-tetramethyl-	−6.402	−32.184
631966	*N*-(Pentafluorobenzylidene)-beta,3,4-tris(trimethylsiloxy) phenylethylamine	−6.357	−39.166
631978	Glycine, *N*-methylsulfonyl-*N*-(4-chloro-2-methylphenyl)-, 4-benzylpiperidide	−6.272	−33.443
621281	3-(*N*-Methylanilino)-2-(triphenylsilyl)-2-cyclobuten-1-one	−6.092	−31.995
3108	Dipyridamole	−5.693	−49.056
8117	Di(hydroxyethyl)ether	−5.642	−27.889
6426589	2-(4-Methoxyphenyl)amino-5,6-difluoro-3-trifluoromethyl-4-heptafluoropropylthiopyridine	−5.503	−29.084
11776	Triphenylphosphine	−5.489	−19.828
624972	4-Hydroxy-3-methoxyphenylacetic acid, ethyl ester, PFP	−5.466	−28.284
5379882	*trans*,*trans*-1,1′-(*m*-Phenylene)bis(3-(*p*-(methylthio)phenyl)-2-propen-1-one)	−5.448	−41.081
634764	Xanthine, 8-[3-iodocyclopentyl]-1,3-dipropyl-	−5.418	−36.475
348969051	diethyl 2-(1-hydroxyethylidene)malonate	−5.214	−30.149

## Data Availability

The data presented in this study are available on request from the corresponding author.

## Supplementary Materials

The following supporting information can be downloaded: Table S1: Compound extracted from *B. diffusa* retrived from GCMS.

## Sample Availability

Samples of the compounds are not available from the authors.
