# Supplementary material for: Evaluation of Nutritional Substances and Investigation of Antioxidant and Antimicrobial Potentials of Boerhavia diffusa with in Silico Molecular Docking
Source: Molecules. 2022 Feb 14;27(4):1280. doi: 10.3390/molecules27041280 (PMC8880713; doi:10.3390/molecules27041280)
Supplement: Supplementary file 1 [file molecules-27-01280-s001.zip › molecules-1546310-supplementary.pdf]

Table S1. Compound extracted from *B. diffusa* retrived from GCMS.

Sl No	PUBCHEM_COMPOUND_CID	Compound Name
1	6329967	1,3,5,7,9-Pentaethylbicyclo[5.3.1]pentasiloxane.1
2	632001	Piperazine-1-ethanol, 4-(2-diethylaminosulfonyll-4-nitrophenyl)-.1
3	631968	(-)-Epinephrine, 3TMS derivative.1
4	348969051	((1-Hydroxyethylidene)malonic acid diethyl ester).1
5	10917006	(25R)-22alpha,26-Epithiofurost-5-en-3beta-ol.1
6	5364290	(Z)-Difluorodiazene.1
7	634748	[trans(trans)]-4-(4-Pentylcyclohexyl)-4'-(4-propylcyclohexyl)biphenyl.1
8	523469	1-(Trihexylsilyloxy)decane.1
9	15373	1-Chloro-1-fluoroethane.1
10	31370	1-Chloro-2-propanol.1
11	111843	1-Ethoxyhexane.1
12	236167	1-Hydrazino-2-propanol.1
13	7900	1-Methoxy-2-propanol.1
14	136819	1,2,3-Triphenylazulene.1
15	635680	1,2,4,5-Tetraaza-3,6-disilacyclohexane, 3,6-dimethyl-1,2,4,5-tetraphenyl-3,6-divinyl-.1
16	6329965	1,3,5,7-T etraethyl-1-ethylbutoxysiloxycyclotetrasiloxane.1
17	635668	1,3,5,7,9,11-Hexaethyl-5,9-dimethoxytricyclo[5.5.1.1(3,11)]hexasiloxane.1
18	6329968	1,3,5,7,9,11-Hexaethylcyclohexasiloxane.1
19	588499	1,4-5,8-Diethanonaphthalene, 1,4,5,8,9,9,10,10,11,11,12,12-dodecafluoro-1,4,4a,5,8,8a-hexahydro-2,3-dimethyl-.1
20	635681	2-(2-Hydroxy-4-octyloxyphenyl)-5-(4-octyloxyphenyl)pyrimidine.1
21	6426589	2-(4-Methoxyphenyl)amino-5,6-difluoro-3-trifluoromethyl-4-heptafluoropropylthiopyridine.1
22	10898	2-Bromoethanol.1
23	6568	2-Butanol.1
24	29420	2-Ethoxy-1-propanol.1
25	634762	2-Ethoxycarbonyl-3-methyl-4-azafluorenone, 2-fluorenylimime.1
26	9737	2-Fluoroethanol.1
27	6420594	2-Fluoroformyl-3,3,4,4-tetrafluoro-1,2-oxazetidine.1
28	8019	2-Methoxyethanol.1
29	12252	2-Nitroethanol.1
30	20483	2,2'-Trithiobisethanol.1
31	262	2,3-Butanediol.1
32	520778	2,3-Dihydroxybenzoic acid.1
33	9570661	2,3-Dioxobutyric acid 2-methyloxime ethyl ester.1
34	1491	2,4-Dihydroxybenzoic acid.1
35	292429	2,5-Bis(5-tert-butyl-2-benzoxazolyl)thiophene.1

36	3469	2,5-Dihydroxybenzoic acid.1
37	9338	2,6-Dihydroxybenzoic acid.1
38	101642136	2',3'-Didehydro-3-de(methoxycarbonyl)-2'-deoxyvobtusine.1
39	534023	2H-Benzocyclohepten-2-one, 3,4,4a,5,6,7,8,9-octahydro-.1
40	621281	3-(N-Methylanilino)-2-(triphenylsilyl)-2-cyclobuten-1-one.1
41	631965	3,4-Dihydroxymandelic acid, ethyl ester, tri-TMS.1
42	530207	3,4-Dihydroxymandelic acid.1
43	530365	3,4-Dihydroxyphenylglycol, 4TMS derivative.1
44	6951097	3,4-Dihydroxyphenylglycol.1
45	5378203	4-(4-Ethoxycarbonylbuta-1,3-dienyl)-1-methyl-2,5-diphenyl-1H-pyrrole-3- carboxylic acid, ethyl ester.1
46	624972	4-Hydroxy-3-methoxyphenylacetic acid, ethyl ester, PFP.1
47	631974	4-Triisopropylsilyloxyhexadecane.1
48	632009	4-Tripropylsilyloxyhexadecane.1
49	635646	4,4'-Bis[(4-chlorophenyl)sulfonyl]-1,1'-biphenyl.1
50	5366461	5-Carbethoxysuccinylacetone, O,O,O-tris(trimethylsilyl)-..1
51	632028	5,12d-Ethano(furo[2,3,4-mn]oxepino[2,3,4-ed]anthracen-9-ol-2-one), 6-methyl-12acetoxy-2a,3,4,4a,5,7,8a-octahydro-.1
52	11144488	entry.1
53	11144488	5alpha-Cholestan-3-one ethylene acetal.1
54	22296474	5alpha-Cholestan-19-oic acid, 2beta-methoxy-.1
55	536275	9-Desoxo-9-xi-hydroxy-3,7,8,9,12-pentaacetate ingol.1
56	8117	111-46-6.1
57	7765	Acetal.1
58	457194	alpha-Conidendrin.1
59	86472	alpha-Tocopherol acetate.1
60	632030	alpha.-Lumicolchicine.1
61	597057	alpha.-T ocopherol-.beta.-D-mannoside.1
62	632012	Amodiaquine TMS derivative.1
63	6957671	Amodiaquine.1
64	635660	Androstane, 17-(2(5H)-oxofuran-4-yl)-3-(t-butyldimethylsilyloxy)-14-(trimethylsilyloxy)-.1
65	6425581	Arsine, (oxo)phenyl-, tetramer-.1
66	631967	Benzeneacetic acid, alpha,3,4-tris[(trimethylsilyl)oxy]-, methyl ester.1
67	530207	Benzeneacetic acid.1
68	1567	beta-Mercaptoethanol.1
69	6713941	beta.-Lumicolchicine.1
70	94900	Bis(2-methoxyethyl) carbonate.1
71	554502	Bis[tert-butyl(dimethyl)silyl] sebacate.1
72	9623	Carbonyl fluoride.1
73	635667	Cholest-2-eno[2,3-b]indole.1

74	11987212	Cobalt, bis(.eta.-5-piperidinylcyclopentadienyl)-.1
75	519601	Cyclodecasiloxane, eicosamethyl-.1
76	11172	Cyclononasiloxane.1
77	11170	Cyclooctasiloxane, hexadecamethyl-.1
78	10913	Cyclopentasiloxane, decamethyl-.1
79	634765	Dibenz[a,h]anthracene, 5,12-diphenyl-.1
80	84166	Diethoxymethyl acetate.1
81	8146	Diethylene glycol monoethyl ether.1
82	3108	Dipyridamole.1
83	2117	DL-alpha-Tocopherol acetate.1
84	519601	Eicosamethyl-cyclodecasiloxane.1
85	22296881	Estra-1,3,5(10)-trien-17-one, 3,4-bis[(trimethylsilyl)oxy]- (CAS).1
86	22296483	Estra-1,3,5(10)-triene-7,17-dione, 3-[(trimethylsilyl)oxy]-.1
87	702	Ethanol.1
88	80169	Ethyl diethoxyacetate.1
89	95630	Ethyl ethoxyhydroxyacetate.1
90	8025	Ethyl formate.1
91	7344	Ethyl lactate.1
92	9620	Fluoroethane.1
93	3045276	Galbulin.1
94	387164927	gamma.-LUMICOLCHICIN.1
95	13589	Glycidyl methyl ether.1
96	631978	Glycine, N-methylsulfonyl-N-(4-chloro-2-methylphenyl)-, 4-benzylpiperidide.1
97	3776	Isopropyl alcohol.1
98	631961	Isoproterenol tri-TMS derivative.1
99	878	Methanethiol.1
100	136386	Methyltartronic acid.1
101	631966	N-(Pentafluorobenzylidene)-beta,3,4-tris(trimethylsiloxy) phenylethylamine.1
102	553889	N-(Trifluoroacetyl)-O,O',O''-tris(trimethylsilyl)norepinephrine.1
103	542733	N-Methoxyformamide.1
104	641324	Nickel, bis(1,3-diphenyl-1,3-propanedionato)-.1
105	524460	NOREPINEPHRINE-TETRA TMS.1
106	951	Norepinephrine.1
107	4113	O-Methylhydroxylamine
108	319242166	Phenethylamine, N-methyl-.beta.,3,4-tris(trimethylsiloxy)-.1
109	553894	Phosphoric acid, bis[2,3-bis(trimethylsiloxy)propyl] trimethylsilyl ester.1
110	6424160	Phthalic acid, monoamide, N,N-diheptyl, pentyl ester.1
111	631963	Propanoic acid, 3-[bis[(trimethylsilyl)oxy]phosphinyl]-, trimethylsilyl ester.1

112	1030	Propylene glycol.1
113	634168	Pyrrolidine, 4-((N,N-dimethylcarbamoyl)benzyl)triphenylacetyl-.1
114	11776	Rhodium, carbonyl(.eta.5-2,4-cyclopentadien-1-yl)(triphenylphosphine)-.1
115	439846	S-1,2-Propanediol.1
116	5192	Sebacic acid.1
117	22296892	Silanamine, N-[(17beta)-3,17-bis[(trimethylsilyl)oxy]estra-1,3,5(10)-trien-2-yl]-1,1,1-trimethyl-.1
118	530365	Silane.1
119	5281	Stearic acid.1
120	632005	Terbutaline, N-trifluoroacetyl-o,o,o-tris(trimethylsilyl)deriv..1
121	167767	Tetracosamethyl-cyclododecasiloxane.1
122	5380044	trans-4-Nitro-4'-(octadecyloxy)chalcone.1
123	5379882	trans,trans-1,1'-(m-Phenylene)bis(3-(p-(methylthio)phenyl)-2-propen-1-one).1
124	5379880	trans,trans-1,1'-(p-Phenylene)bis(3-(p-(methylthio)phenyl)-2-propen-1-one).1
125	31214	Triethyl orthoformate.1
126	5363841	Tris(3-benzyl-2,4-pentanedionato)chromium(iii).1
127	20353	Vitamin e succinate.1
128	14985	Vitamin E.1
129	634764	Xanthine, 8-[3-iodocyclopentyl]-1,3-dipropyl-.1
